# Hip resurfacing: expectations and limitations

**DOI:** 10.3109/17453670903350131

**Published:** 2009-10-01

**Authors:** K De Smet, A Calistri

**Affiliations:** Department of Orthopaedics, ANCA Medical CentreKrijgslaan 181, BE-9000 GhentBelgiumdr.calistri@ancaclinic.it

*Sir*—We read with interest the article by Pieter T. J. Spierings (2008) pertaining to the hip resurfacing expectations and limitations. We would like to make some comments and discuss some of the conclusions of the article:

1. For the conservative approach of the acetabular bone stock, from our experience we do not agree that more bone is removed in hip resurfacing (HRA) than in total hip arthroplasty (THA). The author does not take into account the difference in gender in the indications of both procedures. That a small survey about sales of prostheses done among the largest distributors in the Netherlands showed that there would be more excessive bone loss in resurfacing is possible, but it has been well established in all papers about HRA and THA, that the majority of patients are male in HRA (70%), female in total hip (70%) (Buergi and Walter 2007). In general the cup size in females is about 6 to 8 mm smaller than the size in male patients, which supports our point that there is not a difference between the 2 procedures even in the Netherlands.

There is also a difference in bone removal of the acetabulum in different implants because of different instrumentation, techniques and design, but this has not yet been shown to be statistically significant.

Vendittoli in a prospective, randomized study compared conservation of acetabular bone after THA and HRA of the hip (Venditolli et al. 2006). The results suggested that removal of bone on the acetabular side in HRA was comparable with that of THA, the mean or median diameters of the last reamer used or the mean size of the acetabular component (54.90 mm (44 to 64) for resurfacing arthroplasty and 54.74 mm (48 to 62) for THR, p = 0.770. The same results were also seen in other studies (Naal et al. 2009). Moonot showed hip resurfacing acetabular components was 2.03 mm less than that of the acetabular components in the uncemented total hip replacements (p < 0.0001) (Moonot et al. 2008).

We will not deny that an excessively large cup is never done in resurfacing, but this is seen as a mistake in the resurfacing technique as is also the high abduction angle in cup position.

In revision of HRA it was reported that revision of HRA may be performed successfully with a minimal increase in bone loss (Ball et al. 2007, McGrath et al. 2009). In our study based on (42) we show that the average increase in cup diameter after revision is only 1.4 mm in the hips that needed cup exchange.(De Haan et al. 2008)

2. That the range of motion is “clearly compromised” is not true. This discussion is not so simple and depends mainly on the head neck ratio we can find as well in THA where the head diameter is the most important, in HRA where the head neck ratio is important as is also the surgical technique. If a resurfacing is well done with the correct head size, all osteophytes are removed, and a correct removal of bump on the neck or conflicting bone, the same ROM as a normal hip should be seen.

The proof used in this article with a CAD model derived from CT scans, or composite femurs and pelvises can not in any way be in conflict with daily arthroplasty practice where this problem is not encountered. In some clinical studies greater improvements in hip extension and abduction moment were found in HRA, indicating typical loading of the hip, compared to THA. Shrader in his pilot study showed that HRA group achieved greater hip extension through the movement cycle compared to the THA group, which maintained reduced (p = 0.01) hip extension angle (Shrader et al. 2009).

That impingement problems and reduction of ROM never occurs in HRA we will not state, but this problem does not have a high incidence/prevalence.

In the literature a variety of other complications related to HRA can be found, including metallosis, raised metal ion levels, aseptic lymphocytic vasculitis associated lesions (ALVAL), pseudotumors, clicking, squeaking, and nerve palsy (Back et al. 2005, Lachiewicz 2007). Mabilleau give in this issue of Acta an overview of the literature on biological responses to metal-on metal HRA. They found an increasing number of case reports on periprosthetic soft-tissue masses and osteolysis as a response to elevated metal ion levels. (Mabilleau et al. 2008) The increased concentration of metal particles in the joint space of HRA could lead to a T lymphocyte-mediated hypersensitivity reaction (Type IV). The authors express their concerns about the risks of long-term exposure to metal ions. An increased risk of developing lymphoma in patients with chronic inflammatory disease who undergo metal-on-metal arthoplasty has recently been considered (Lidgren 2008).

The same issues can be found with metal-on-metal total hips, especially in the current era of large diameter jumbo heads. Those are rare metal-on-metal problems, not simply HR problems.

Varus placement of the femoral component leads to higher levels of stress and increases the probability of failure (Beaulé and Poitras 2007, Radcliffe and Taylor 2007, Lazarinis et al. 2008). Cup anteversion greater than 25% or cup abduction less than 45% can result in impingement and increased wear. The safe zone for cup and head positioning is smaller in HRA than in THA, and deviations are less forgiving.

Again, this is a big diameter MoM problem – there is clear evidence that component design is an important determinant of component wear when the implant is malpositioned, so not all HRAs will suffer from this problem to the same extent.

Stress Shielding does occur to some extent in all Hip replacements but the degree and clinical consequences are highly variable according to confounding factors including initial bone stock, vascularity, fixation and biomechanical integrity of the construct. Analysis of long-term retrieved specimens (up to 23 years) shows that this is not inevitable or clinically consequential in many well performed HRAs. Indeed, these long terms specimen often show remarkable remodelling and adaptation, even in female patients (Kordi and McMinn 2009).

Dr Spierings points out that Hing et al. “measured more than 10% thinning in 28% of his patients.” which sounds alarming. However, in her paper, Dr Hing concluded that “narrowing of the femoral neck which is found with the Birmingham hip resurfacing arthroplasty is in most cases associated with no adverse clinical or radiological outcome up to a maximum of six years after the initial operation” (Hing et al. 2007).

We agree that Patients are best served with proven designs that have proven long-term outcome.” With greater than 10 year follow-up in the first two HRA designs, and with excellent results in a difficult group of patients, there are proven designs of HRA available for surgeons willing to undergo the training to become specialist resurfacing surgeons.

We do also agree that easy resurfacing does not exist, even in experienced hands. Hip resurfacing arthroplasty only finds its place in high volume centres and experienced surgeons. A heart transplant is not done by a general surgeon, but only in a specialised centre.
