# Supplementary material for: Hip resurfacing: expectations and limitations
Source: Acta Orthop. 2009 Oct 1;80(5):625–7. doi: 10.3109/17453670903350131 (PMC2823340; doi:10.3109/17453670903350131)
Supplement: Related texts

# Sub article

**Published:** 2009-11-17

**Authors:** Pieter TJ Spierings

**Affiliations:** Madoerastraat 24 6524LH NijmegenThe Netherlandspspierings@spierings.biz

*Sir*—Thanks for the letter with comments from Drs. De Smet and Calistri in which they discuss various topics of my guest editorial.

*1. More acetabular bone loss in HRA*. Data obtained from Dutch distributors showed a difference of approximately 6 mm between the cup diameter of a total hip arthroplasty (THA) and a hip resurfacing arthroplasty (HRA). De Smet and Calistri claim this difference is due to a difference in sex ratio between THA and HRA. They refer to an article using data from the 2005 Australian National Joint Replacement registry. In this registry the male/female percent ratio for HRA is 44/56 and for THA 73/27. The difference in the average acetabular cup diameter between males and females is 4–5 mm (Moonot et al. 2008, Naal et al. 2009). Simple calculations then shows that the difference in sex ratio between THA and HRA accounts only for 29% × 4.5 mm = 1.3 mm diameter difference. This means that the remaining 4.7 mm or 78% of the difference in cup diameter between THA and HRA must be contributed to the surgical procedure.

Publications have shown differences between THA and HRA cup diameter. Vendittoli et al. (2006) found no difference in cup diameter. Moonot et al. (2008) found in an age and sex matched cohort no difference in cup diameter for men, and for women a 2 mm smaller cup diameter in HRA, than for uncemented THA. Naal et al. (2009) not only matched for age and sex but also for height and weight, which are associated with the cup diameter. They found that HRA required, for both men and women, a 2 mm larger acetabular component than uncemented THA. Loughead et al. (2006) found a 5 mm larger cup diameter for HRA than for THA.

Certainly surgical technique and skill plays a major role in the outcome of these studies. In the Netherlands no centers exist which are fully committed to resurfacing arthroplasty and taking into account that only 6% of all hip patients qualify for a HRA it is clear that only few surgeons will have the opportunity to gain sufficient experience. Less experienced surgeons will try to avoid notching to decrease the risk of femoral neck fracture and will probably use a larger femoral component than an experienced surgeon. Therefore it may be expected that acetabular bone loss for HRA will be higher in a nationwide survey than presented in studies of single high-volume centers.

*2. Compromised range of motion.* There is no doubt that the natural femoral neck diameter in HRA is larger than the prosthetic neck diameter in THA. With a given head diameter size this results in a decreased range of motion (ROM) in flexion and combined flexion-adduction- endorotation movements. I agree that in most cases the decreased ROM will not be noticed clinically and that a sufficient ROM can be achieved. In some cases however it may lead to impingement and subluxation which for a metal on metal bearing is most undesirable. Subluxation will lead to increased wear and metallosis.

3. *Metal on metal.* De Smet and Calistri state that many of the complications and disadvantages related to HRA are not caused by HRA itself but are caused by the metal on metal bearing surfaces. This observation is correct. Unfortunately metal on metal is the only bearing material available for resurfacing prostheses. In many cases metal on metal is an excellent choice with a successful long-term outcome. In some cases, in particular when component positioning and range of motion are critical, metal on metal is a less good choice. Metal on metal is very unforgiving as soon as circumstances become less from optimal. Metal on metal impingement will cause excessive wear as does subluxation. Steep cup positioning will lead to rim loading and stripe wear. The combination of critical positioning, reduced ROM and metal on metal is not ideal, but metal on metal is the only choice for HRA. Any other bearing material would need a thicker acetabular cup wall thickness and therefore a larger cup diameter.

De Smet and Calistri work in a clinic fully specialized in HRA. In such a an ideal setting there is no learning curve and type of implant, instrumentation, bone preparation and cementing technique can be optimized. Their long term survival rate surely must be better than the numbers of a national register. In the 2008 Australian Registry, presenting the results of 2007, the use of HRA has decreased for the second year. Together with an increasing number of all primary hips, the percentage of resurfacing has decreased to 7%. The revison rate for this selected patient group remains high. The 1-year revision rate for HRA is 1.9% compared to 1.1% for cemented THA. The 7-year revision rate is 5.4% for HRA compared to 3.8% for cemented THA. HRA has a significantly higher revision rate compared to THA with a hazard ratio (adjusted for sex and age) of 1.4. Up to now there is no evidence that THA can improve the short-, medium- or long-term results obtained with conventional total hips.
